# Health Resorts

**Published:** 1902-08-30

**Authors:** 


### HEALTH RESORTS.

### CHELTENHAM.

There was a time when Cheltenham was known outside its own neighbourhood chiefly as a place to which people went when their livers were out of order. Its fame then depended entirely upon the virtues of its waters. The mildness of its climate, its beautiful situation and surroundings, and its many other advantages as a place of residence, gradually led a large number of Anglo-Indians and others who had lived much in tropical climates to take up their abode there, and thus it happened that besides being a mere health resort it became a social centre of considerable importance. On this was then grafted another development, which has for some time back overshadowed all the rest, namely the great eminence which it has attained as a place of education, at first for boys and then more especially for girls. Indeed, during recent years the fame of the Cheltenham Ladies' College has spread so widely as to have to a large extent swamped all the other associations of the town. Lately, however, with the growing tendency which has shown itself for people to go to watering-places for the special treatment of many disorders, and it may be added with the increasing confidence now reposed by medical practitioners in hydro-therapeutic methods generally, the advantages which Cheltenham offers as a health resort have again come into prominence, and it is to this aspect of the question that we would here more particularly refer.

The wells from which the Cheltenham waters are derived are now chiefly owned by the Corporation of the town, under whose control they are collected, filtered, and offered for sale. These waters are mainly of two kinds. Both of them are saline waters, but one contains a considerable quantity of magnesium sulphate as well as calcium sulphate in addition to its other constituents, and is therefore called the magnesia saline, while the other, which by-the-by is much stronger in sodium chloride than its fellow, contains sodium bicarbonate, calcium carbonate, and magnesium carbonate, and is spoken of as the alkaline saline.

Thus we have two very useful waters, both mildly aperient, alterative and diuretic, both containing a small quantity of iron and also certain quantities of bromides and iodides, but in one of which the dominating characteristic is the presence of sulphate of magnesia, soda, and lime, while in the other the sulphate of soda is combined with carbonates. When aërated the alkaline saline is often drunk at meals, the aëration going far to take away the taste of the sodium sulphate. According to the pamphlet issued by the corporation the salt is just in sufficient quantity to give the water a pleasant flavour, whilst the sulphate of soda is "not sufficient to offend the taste, but enough to stimulate secretion without acting as an aperient." We believe, however, that if taken in full doses an aperient effect is generally obtained, and in case this should not happen, recourse can be had to the so-called Cheltenham Natural Aperient, which consists of the magnesia saline water evaporated down. By the process of evaporation much of the lime is got rid of, and supplied as this water is in a slightly aërated form it provides a decidedly useful and by no means unpleasant morning aperient saline.

In addition to these waters there is a proprietary well, the Cambray Spa, which contains sufficient iron to be regarded as a chalybeate. There can be no doubt about the therapeutic efficacy of the Cheltenham waters, which are prescribed in various gouty and catarrhal affections, but probably their true use is to cut at the preceding disturbance of the liver and digestive organs, the so-called lithæmia, which acts as a predisponent to more definite disease. They must be regarded as "mild" waters which are likely to attain their greatest efficacy among patients who are not too ill to enjoy the social and other advantages with which the beautiful town abounds.

### FALMOUTH.

Falmouth is a place which, for the equability of its climate, is almost unique in the British Isles. Hence its fame as a health resort. Its climate may in many respects be regarded as oceanic, for Cornwall juts so far out into the Atlantic, its coast line is so irregular, the sea is everywhere so near, and the water of that sea is so constantly renewed by the great stream which brings to our western coasts the warmth and sometimes the actual products of the Gulf of Mexico, that while in winter warmth becomes the characteristic of the place, in summer the freshness of sea breezes most predominate. Thus so far as water can influence the climate of land Falmouth is influenced, and that mostly in the direction of equability—coolness in summer heats, warmth when other parts of England shiver. With such natural advantages Falmouth ought to be, and some time will undoubtedly become, one of the leading health resorts of this country. It does but want a steady perseverance in sanitary well doing to bring to itself a most prosperous future.

Falmouth is a town of about 12,000 inhabitants. It is built near the base of a tongue of land which juts out into the sea and divides Falmouth Harbour from Falmouth Bay, the point of this promontory forming the western side of the opening of the broad inlet of the English channel, known as Carrick Roads, which runs up nearly to Truro. The greater part of the town, in fact, all the old town, faces north-east towards the Harbour, which is a large sheet of water. But the growth of the town has carried it over the ridge of the hill, so that some of the newer and residential portion faces the bay and looks out over the English Channel. This portion is well removed from the older and more closely built business part of the town.

The whole question at Falmouth hinges upon sanitation. The beauty of the place, the admirable climate it possesses for the treatment of cases in which it is necessary to obtain protection from the rigours of the English winter, the charming excursions which can be made from it both by land and water, and, so far as the latter are concerned, the fact that excursions by steamer can be had on land-locked sea extending for many miles, all make Falmouth an ideal place for certain cases, while as a summer resort it is all that can be desired.

Unfortunately the prevalence of enteric fever some years ago and an epidemic which occurred in 1899 drew down upon it a very unfavourable report as to its sanitary administration from an inspector sent down by the Local Government Board. Shortly, the report of this official went to show that the general sanitary condition of the borough was in many ways unsatisfactory; that there were defects in house construction, crowded courts, badly paved yards, leaky sewers, faulty house drainage, and objectionable methods of sewage disposal on the foreshore of Falmouth Harbour; that there were opportunities for the pollution of the "gathering ground" from which the water supply of the town was drawn, that the filtration of the water was crude and insufficient, that the distributing pipes were liable to choke from peaty matters in the water, that the pressure on the high service was insufficient, that the supply was periodically intermitted, and that there were reasons for believing that one thing at least which had to do with the epidemic which began in August, 1899, was insucking of infectious matter into the water mains during periods of intermission of supply. In view of the state of affairs thus brought to light, we have made inquiries as to the steps taken by the corporation to remedy the evils referred to and have received the following reassuring statement from the Town Clerk, which will be read with much interest by intending visitors. He says: "Since we received the Local Government Report of 1899 immense improvements have been effected in the drainage of the borough. Two inspectors have been engaged ever since, and every house drain in the borough has practically been tested. If the slightest leakage has been detected, the owner has been called upon to remedy same. Thousands of pounds have been expended in this manner. Drains under houses have been embedded in concrete, the drains properly intercepted, w.c.'s properly flushed, badly-paved yards taken up and repaved, unsanitary houses closed. The dairies and cowsheds thoroughly inspected and put in an up-to-date sanitary condition; an isolation hospital erected by way of precaution, but the town has been so remarkably free from disease that we have had no occasion to use same, and hope we never shall. Factories, workshops, and bakehouses have been put in a thoroughly sanitary condition, and I think we may safely say that no town in England of our size has done so much during the last two and a half years to put the same in a good sanitary condition. The water supply has been attended to and the analyst now states we need wish for no better or purer water."

